# Clinical Remission of an Unresectable Presumptive Haemangiosarcoma in a Dog Treated With Toceranib, Piroxicam, and Propranolol: A Case Report

**DOI:** 10.1002/vms3.71043

**Published:** 2026-06-19

**Authors:** Woo Dae Park

**Affiliations:** ^1^ Department of Veterinary Surgery Pet in Zoo Animal Medical Center Uijeongbu Republic of Korea

**Keywords:** dog, haemangiosarcoma, piroxicam, propranolol, toceranib

## Abstract

Haemangiosarcoma (HSA) is a highly aggressive vascular tumour in dogs, characterised by rapid growth, early metastasis, and poor prognosis despite conventional treatment with surgery and doxorubicin‐based chemotherapy. A 14‐year‐old spayed female Maltese (4.14 kg) was presented with progressive abdominal distension and anorexia. Imaging revealed a large retroperitoneal mass, and computed tomography identified a 12.1 × 8.0 × 8.5 cm heterogeneous tumour. Exploratory laparotomy confirmed the lesion was unresectable, and biopsy findings were most consistent with presumptive haemangiosarcoma (HSA) due to the small, cautery‐affected specimen. Medical therapy was initiated with toceranib phosphate (10 mg every other day), followed one week later by propranolol (0.3 mg/kg twice daily) and piroxicam (0.3 mg/kg once daily). The patient was monitored every 2–4 weeks with physical examination and serial haematology and serum biochemistry. Two months after initiating therapy, the abdominal ultrasonography demonstrated tumour reduction to 4.9 × 2.7 cm, and after 7 months the mass was no longer detectable on radiographic follow‐up imaging (radiography and ultrasonography). Throughout treatment, haematologic and biochemical values remained within reference intervals, no clinically significant adverse effects were observed, and the owner reported resolution of abdominal distension with improved activity. This case demonstrates that combined therapy with toceranib, propranolol, and piroxicam achieved radiographic and clinically sustained remission of unresectable canine presumptive HSA while preserving quality of life. These findings suggest that multimodal therapy targeting angiogenesis and tumour growth pathways may represent a promising alternative strategy for managing this malignancy and warrant further clinical evaluation.

## Graphical abstract

Radiographic remission of a presumptive unresectable haemangiosarcoma treated with toceranib, piroxicam, and propranolol. In a dog with ACVIM Stage B2 myxomatous mitral valve disease where doxorubicin‐based chemotherapy was contraindicated, this multimodal regimen achieved marked tumour reduction and radiographic disappearance.

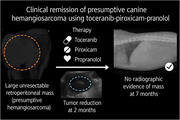

## 1 Introduction

Haemangiosarcoma (HSA) is a common malignant tumour of dogs arising from vascular endothelial cells, accounting for approximately 5–7% of all canine malignancies (Clifford et al. 2000; Wendelburg et al. 2015). It most frequently develops in highly vascularised organs such as the spleen, liver, right atrium, and skin. Splenic HSA is the most prevalent form, typically affecting older, medium‐ to large‐breed dogs. The tumour is characterised by rapid progression, early metastasis, and a high propensity for rupture, often resulting in acute haemorrhage and sudden death (De Nardi et al. 2023).

The current standard of care for canine HSA consists of surgical excision followed by doxorubicin‐based chemotherapy (Clifford et al. 2000; De Nardi et al. 2023; Ogilvie et al. 1996; Wendelburg et al. 2015). Despite this approach, prognosis remains poor, with median survival times of only 1–3 months after surgery alone and 5–9 months with adjuvant chemotherapy (Clifford et al. 2000; De Nardi et al. 2023; Ogilvie et al. 1996; Wendelburg et al. 2015). Moreover, chemotherapy‐associated toxicities such as gastrointestinal upset and myelosuppression can significantly compromise patient quality of life. These limitations underscore the urgent need for more effective and better‐tolerated alternatives (Smith 2003).

Novel therapeutic approaches under investigation include targeted therapy, immunomodulation, and drug repurposing. Toceranib phosphate, the first oral tyrosine kinase inhibitor (TKI) approved for veterinary use, inhibits VEGFR, PDGFR, and KIT signalling – pathways essential for angiogenesis and tumour proliferation (Berger et al. 2018; Gardner et al. 2015; London et al. 2003). Initially licensed for mast cell tumours, toceranib has demonstrated activity against various sarcomas, including HSA (Berger et al. 2018; Gardner et al. 2015; London et al. 2003). Given the tumour's dependence on angiogenesis, toceranib is considered a rational therapeutic option; however, most clinical reports describe disease stabilisation rather than complete remission (Gardner et al. 2015).

Piroxicam, a non‐steroidal anti‐inflammatory drug (NSAID), is commonly prescribed for analgesia and inflammation control but also exerts antitumour effects through cyclooxygenase inhibition. In canine transitional cell carcinoma, piroxicam has prolonged survival when used alone or in combination with chemotherapy (Knapp et al. 1992; Lana et al. 2007). Experimental studies also suggest pro‐apoptotic and antiproliferative effects in HSA cells, and its affordability, oral administration, and relatively mild toxicity profile support its use as an adjunctive therapy.

Propranolol, a non‐selective β‐adrenergic blocker widely used in human medicine for infantile haemangiomas (Léauté‐Labrèze et al. 2008), reduces angiogenesis by down‐regulating VEGF expression, inducing vasoconstriction, and suppressing endothelial proliferation. Preclinical mechanistic studies have demonstrated that propranolol exerts antiproliferative effects in vascular sarcoma cell lines and enhances doxorubicin sensitivity by altering lysosomal drug sequestration and efflux pathways (Saha et al. 2021). Early clinical experience remains limited; a small case series involving five dogs with stage 3 haemangiosarcoma reported clinical benefit when propranolol was combined with anthracyclines (Terauchi et al. 2023). However, findings across these small‐scale reports have been heterogeneous, and no controlled clinical studies have yet confirmed a consistent therapeutic effect, although propranolol remains an appealing candidate for incorporation into multimodal oncologic protocols.

Collectively, toceranib, piroxicam, and propranolol act on complementary mechanisms converging on the tumour–vascular axis, suggesting potential synergy when used in combination. Given the biologic heterogeneity of HSA and the frequent challenges in obtaining large, high‐quality biopsy samples – especially from retroperitoneal lesions – some cases require clinical management based on a presumptive diagnosis rather than definitive histopathology. This context is relevant to the present report.

This report describes a rare case of an unresectable retroperitoneal mass most consistent with presumptive canine haemangiosarcoma that achieved a sustained radiographic remission following multimodal therapy with toceranib, piroxicam, and propranolol. Unlike previous studies that have largely documented disease stabilisation, this case demonstrated radiographic disappearance of the tumour and durable clinical improvement, highlighting the potential of this regimen as a promising alternative for managing aggressive vascular neoplasms in dogs despite diagnostic and imaging limitations.

## 2 Case Presentation

A 14‐year‐old spayed female Maltese (4.14 kg) was presented with a several‐week history of progressive abdominal distension and reduced appetite. Physical examination revealed a firm, cranially located abdominal mass. Mucous membranes were pink, capillary refill time was normal, and no respiratory distress was evident. The dog had ACVIM Stage B2 myxomatous mitral valve disease (MMVD) but remained clinically stable, with no signs of congestive heart failure at presentation.

Baseline monitoring – including heart rate, respiratory rate, body temperature, non‐invasive blood pressure, and pulse oximetry – was instituted. Systolic blood pressure remained between 120–150 mmHg throughout hospitalisation. Because of the large abdominal mass and suspected risk of haemorrhage, the patient was placed on restricted activity and closely monitored while diagnostic imaging was completed.

**FIGURE 1:**
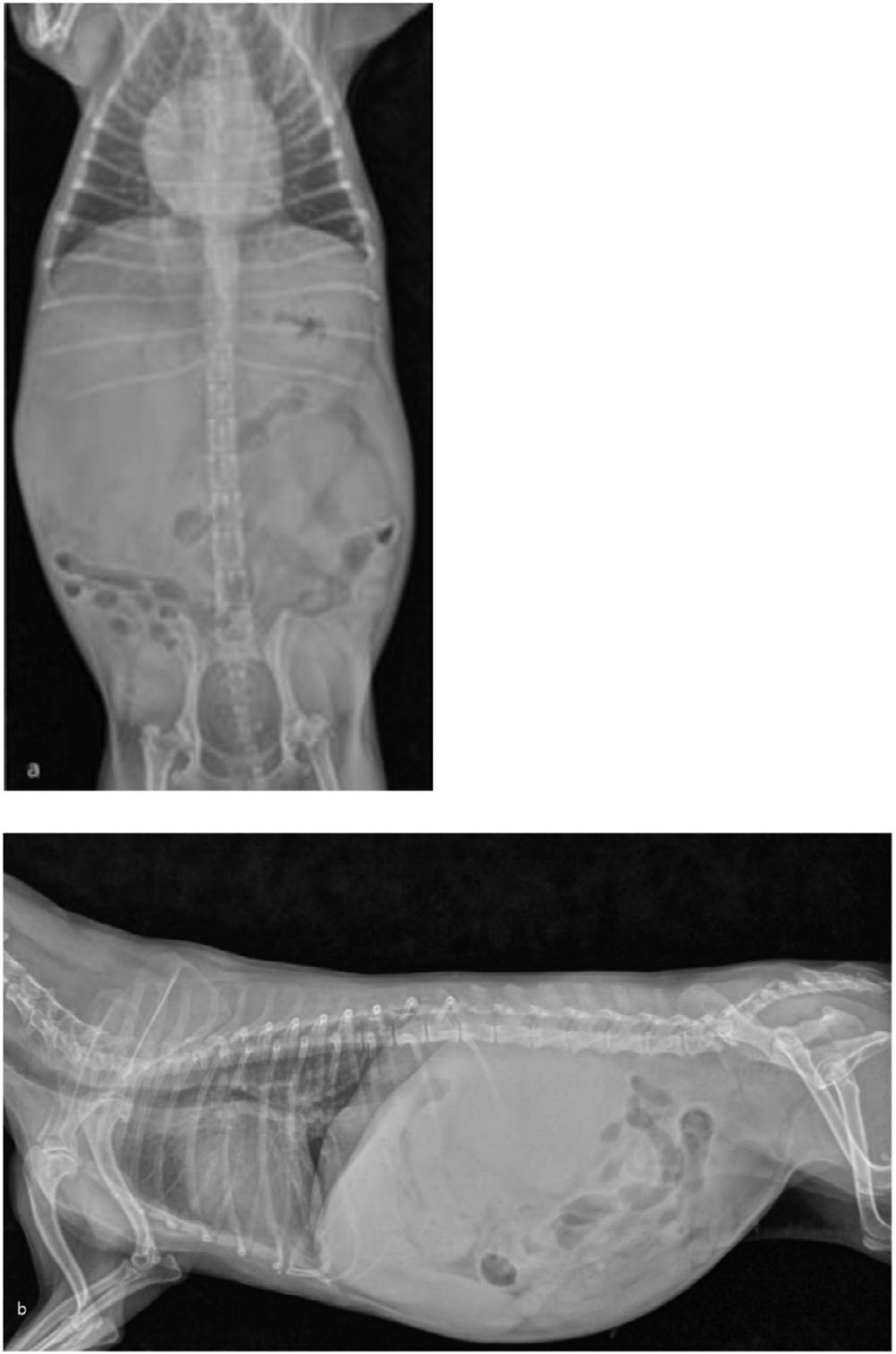
(a) Ventrodorsal view showing a large soft‐tissue opaque mass in the right cranial abdomen, displacing adjacent abdominal viscera. (b) Right lateral view demonstrating cranial abdominal enlargement with poor serosal detail and displacement of intestinal loops, consistent with a space‐occupying lesion.

**FIGURE 2:**
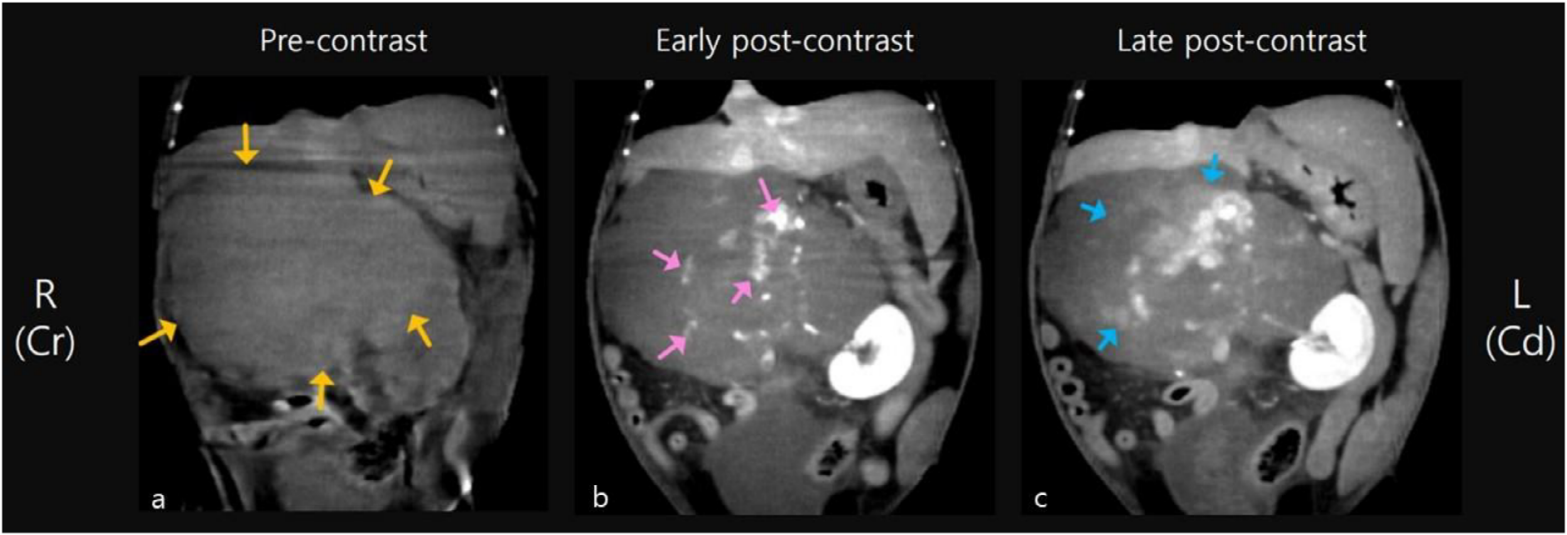
Contrast‐enhanced computed tomography (CT) of the abdomen at initial diagnosis, dorsal plane, soft‐tissue window. (a) Pre‐contrast image showing a large retroperitoneal mass (12.1 × 8.0 × 8.5 cm) of homogeneous soft‐tissue attenuation (yellow arrows). (b) Early post‐contrast image revealing heterogeneous enhancement with focal regions of strong contrast uptake (pink arrows). (c) Late post‐contrast image demonstrating progressive contrast distribution into surrounding parenchyma (blue arrows).

Abdominal radiography revealed a soft‐tissue opaque mass (7.2 × 7.0 cm) in the right cranial abdomen, displacing adjacent organs (Figure 1). Ultrasonography demonstrated a heterogeneous, predominantly hypoechoic retroperitoneal mass near the right kidney with scant peritoneal effusion (Figure 2). Mild enlargement of regional lymph nodes was noted, but all maintained normal echotexture.

**FIGURE 3:**
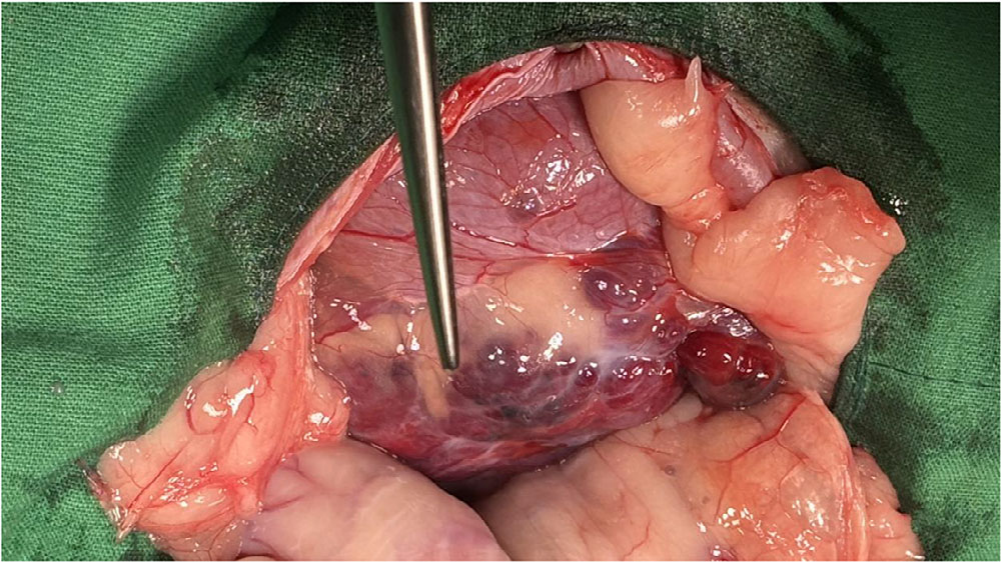
Intraoperative photograph from exploratory laparotomy at initial diagnosis. The retroperitoneal mass shows an irregular surface with multifocal haemorrhagic discoloration. Excision was not possible due to extensive adhesions and haemorrhage risk; a limited biopsy was obtained.

CT was performed under general anaesthesia (butorphanol and glycopyrrolate premedication, propofol induction, sevoflurane maintenance). Pre‐ and post‐contrast images were obtained through early and late phases. A retroperitoneal mass measuring 12.1 × 8.0 × 8.5 cm was identified (Figure 3). Key findings included pre‐contrast homogeneous soft‐tissue attenuation; post‐contrast hypoattenuating parenchyma with multifocal intense enhancement; early‐phase strong focal vascular enhancement; and late‐phase diffuse and peripheral contrast distribution. Additional features included the aorta and major branches adjacent to or traversing the mass without intraluminal invasion; ventral displacement/compression of the caudal vena cava; displacement of the right kidney, stomach, pancreas, and small intestine; mild perilesional fat stranding and peri‐renal fluid; mild hepatic, gastric, and colic lymphadenopathy without abnormal enhancement; cranial mediastinal lymphadenopathy without pulmonary nodules; small splenic mineral nodules (<8 mm), likely benign (extramedullary haematopoiesis or nodular hyperplasia); and small bilateral renal cortical cysts. Impression: Findings were most consistent with a retroperitoneal haemangiosarcoma, although the diagnosis remained presumptive, and histopathologic confirmation was recommended.

**FIGURE 4:**
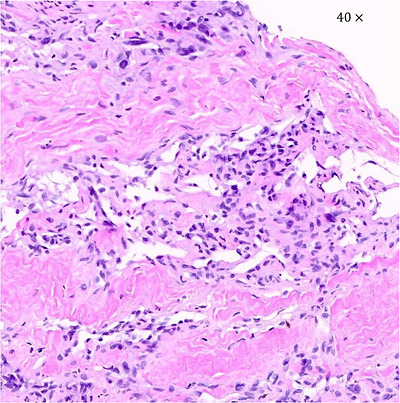
Histopathologic image of the retroperitoneal mass. Haematoxylin and eosin stain, original magnification ×40 (0.216 mm^2^). The biopsy sample consists of polygonal to polyhedral cells embedded in a fibrocollagenous stroma. The image shows a superficial biopsy specimen that is nondiagnostic due to severe cautery artefact; this image is not intended to demonstrate definitive histologic features of HSA but to illustrate the limitations of the available tissue.

Exploratory laparotomy was performed with owner consent. The mass was extremely friable and haemorrhagic, with brisk bleeding occurring even with minimal manipulation. Because of this, complete excision was deemed unsafe. A superficial incisional biopsy was obtained using a LigaSure Small Jaw vessel‐sealing device (Medtronic, Minneapolis, MN, USA) to minimise intraoperative haemorrhage. The device provided immediate sealing of small vessels, allowing controlled sampling. Additional haemostasis included gentle digital pressure, placement of an absorbable gelatin sponge over the biopsy site, and avoidance of deeper penetration into highly vascular regions. The biopsy site was reinforced with omental apposition, and the abdomen was closed routinely (Figure 4). Recovery was uneventful, with no MMVD‐related complications.

**FIGURE 5:**
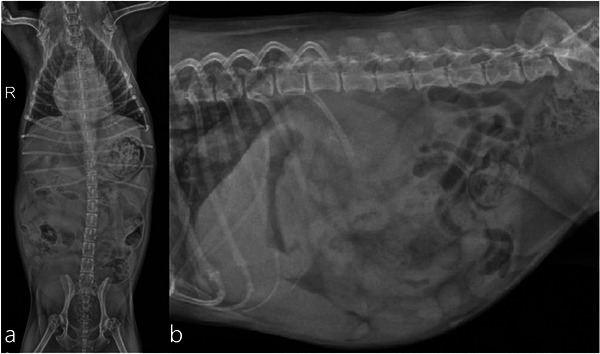
Follow‐up abdominal radiographs after 7 months of therapy. (a) Ventrodorsal view showing no evidence of the previously identified retroperitoneal mass. (b) Right lateral view confirming the absence of a space‐occupying lesion, consistent with complete radiographic remission.

The biopsy contained polygonal to polyhedral cells in a fibrocollagenous stroma with areas of chronic haemorrhage. Architectural and cytologic features could not be reliably evaluated due to extensive cautery artefact and insufficient tissue volume; therefore, the diagnosis remained presumptive. Some cells displayed macrophagic morphology; adrenal cortical origin was excluded. Immunohistochemistry for endothelial markers (CD31, vWF) could not be performed because the biopsy sample was insufficient and markedly affected by thermal (cautery) artefact. Taken together with imaging findings, the mass was considered most consistent with a presumptive haemangiosarcoma (Figure 5).

The patient was hospitalised for 3 days and received multimodal supportive care, including opioid analgesia, antiemetics, antimicrobials, vitamin K1, anticoagulants, and corticosteroids. Cardiovascular parameters and oxygenation remained stable without arrhythmias, respiratory compromise, or worsening of MMVD.

At discharge, toceranib phosphate (10 mg every other day; ∼2.4 mg/kg EOD) was initiated. After one week without adverse effects, propranolol (0.3 mg/kg twice daily) and piroxicam (0.3 mg/kg once daily) were added. Doxorubicin‐based chemotherapy was avoided because the tumour exhibited severe haemorrhagic behaviour, increasing risk of catastrophic bleeding; the patient had ACVIM Stage B2 MMVD, placing her at increased risk for anthracycline‐induced cardiotoxicity; and the owner declined high‐risk chemotherapy given the dog's age and comorbidities. The dog was examined every 2–4 weeks with physical examination, CBC, serum biochemistry, and cardiovascular monitoring. No significant gastrointestinal, renal, haematologic, or haemodynamic adverse effects were observed. Despite MMVD and β‐blocker therapy, heart rate, systolic blood pressure, and clinical status remained stable, and no decompensation occurred.

**FIGURE 6:**
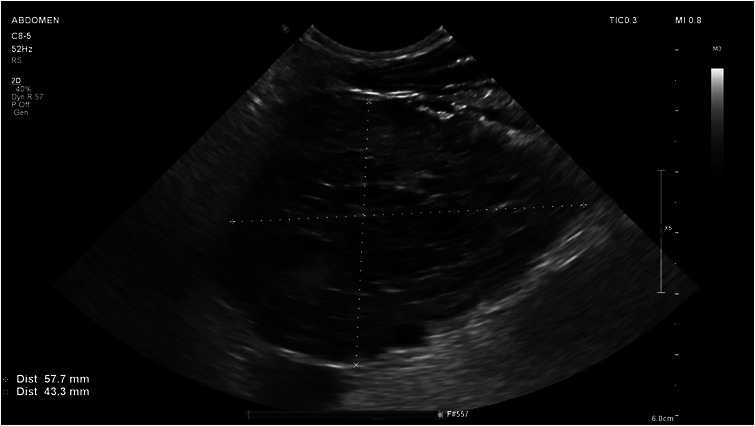
Abdominal ultrasonography at initial diagnosis. (a) Transverse view showing a large heterogeneous hypoechoic mass (5.8 × 4.3 cm) with poorly defined margins. (b) Longitudinal view adjacent to the right kidney demonstrating the same heterogeneous mass, with a small amount of peritoneal effusion.

**FIGURE 7:**
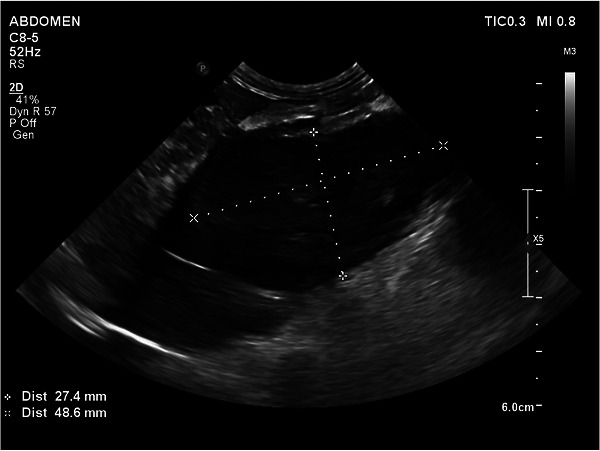
Abdominal ultrasonography after 2 months of therapy. The cranial abdominal mass measured approximately 4.9 × 2.7 cm, markedly reduced compared with the initial study, indicating partial therapeutic response.

At 2 months, ultrasonography demonstrated reduction of the mass to 4.9 × 2.7 cm (Figure 6), accompanied by resolution of abdominal distension and improved activity. At 7 months, abdominal radiographs showed no detectable mass (Figure 7). As follow‐up consisted only of radiography and ultrasonography, confirmation of complete remission by CT was not possible; therefore, findings represent radiographic remission. Throughout follow‐up, laboratory values stayed within reference ranges, appetite and activity were normal, and MMVD remained clinically stable with no signs of congestive heart failure.

## 3 Discussion

Canine haemangiosarcoma (HSA) carries a guarded to poor prognosis even with aggressive therapy, as most dogs experience rapid progression and early dissemination despite splenectomy and doxorubicin‐based chemotherapy (Clifford et al. 2000; Wendelburg et al. 2015). Durable responses in unresectable visceral forms are exceedingly rare. Recent oncogenomic studies further highlight the biological heterogeneity of HSA, identifying distinct molecular subsets defined by mutations in PIK3CA, NRAS, and TP53, which may influence tumour behaviour and therapeutic responsiveness (Paoloni and Khanna 2008; Wang et al. 2020; Wong et al. 2021).

In the present case, prognostic uncertainty was compounded by significant diagnostic constraints. The mass demonstrated extreme vascular friability, resulting in brisk intraoperative haemorrhage that allowed only a superficial, limited biopsy using a LigaSure device. The resulting tissue was small and markedly affected by thermal artefact, preventing assessment of endothelial atypia, vascular channel formation, or mitotic activity, and precluding immunohistochemistry. Consequently, the diagnosis must be regarded as presumptive rather than definitive. This limitation is essential when interpreting the magnitude of the clinical response observed.

Although doxorubicin remains the standard chemotherapeutic agent for canine HSA, its use was avoided in this patient for several medical and safety reasons. First, the dog had ACVIM Stage B2 myxomatous mitral valve disease with structural remodelling (LA/Ao 1.86; LVIDdN 2.0), which substantially increases susceptibility to anthracycline‐induced cardiotoxicity. Second, the tumour exhibited pronounced haemorrhagic fragility intraoperatively. Because doxorubicin can induce thrombocytopenia and coagulopathy, initiating anthracycline therapy in a patient with severe baseline bleeding risk was considered unsafe. Third, the dog's advanced age (14 years) and small body weight (4.14 kg) increased the likelihood of clinically significant gastrointestinal and haematologic toxicity. Additionally, the owner declined high‐risk chemotherapy given the comorbidities and procedural risks. Collectively, these factors supported the selection of a non‐anthracycline multimodal regimen focused on antiangiogenic and stromal‐modulating mechanisms while minimising systemic toxicity.

Each therapeutic agent influences complementary biological pathways relevant to HSA pathogenesis. Toceranib inhibits VEGFR, PDGFR, and KIT, suppressing angiogenic and proliferative signalling; most reported responses involve disease stabilisation rather than regression (Gardner et al. 2015; London et al. 2003). Piroxicam reduces prostaglandin synthesis via COX inhibition and promotes apoptosis and tumour microenvironment modulation (Knapp et al. 1992; Lana et al. 2007). Propranolol suppresses VEGF expression, blocks β‐adrenergic signalling, and enhances chemosensitivity in vitro by altering lysosomal drug sequestration (Saha et al. 2021). A small case series suggested potential benefit when combined with anthracyclines (Terauchi et al. 2023). Together, these mechanisms provide a biologically plausible rationale for combination therapy, although no studies to date have evaluated this specific triple regimen in vivo. Therefore, any interpretation of pharmacologic synergy remains speculative and hypothesis‐generating rather than confirmatory.

This case demonstrates several clinically meaningful findings. First, most literature describes stabilisation with toceranib, whereas complete radiographic disappearance of disease, as observed here, is rare. Second, the patient remained clinically stable for at least 7 months, exceeding expected outcomes for unresectable visceral HSA treated without anthracyclines. Third, no significant gastrointestinal, haematologic, renal, or cardiovascular toxicity was observed. Notably, propranolol was well tolerated despite the patient's ACVIM Stage B2 MMVD, with no evidence of decompensation. Fourth, severe intraoperative haemorrhage necessitated a limited biopsy using a LigaSure device, resulting in thermal artefact that prevented definitive diagnosis and emphasised the need for cautious interpretation.

Possible biological explanations for the unusually robust response include a molecular subtype highly dependent on VEGF‐driven angiogenesis, enhanced reliance on β‐adrenergic signalling for vascular support, rapid vascular collapse early in treatment leading to disproportionate loss of tumour viability, and innate tumour sensitivity to antiangiogenic stress pathways. These hypotheses remain speculative but align with current understanding of HSA heterogeneity.

Several limitations must be acknowledged. These include the presumptive diagnosis due to limited, cautery‐affected biopsy tissue; inability to perform immunohistochemistry (CD31, vWF) or molecular profiling; restricted follow‐up imaging using radiography and ultrasonography only, preventing confirmation of complete remission; and the single‐case design, limiting generalisability. Future studies should evaluate multimodal antiangiogenic regimens in prospective cohorts, compare non‐anthracycline protocols with standard therapy, and incorporate genomic and immunohistochemical biomarkers to identify responsive HSA subtypes.

## 4 Conclusion

This case describes a dog with an unresectable retroperitoneal mass most consistent with a presumptive haemangiosarcoma that achieved clinical remission, including radiographic disappearance, following multimodal therapy with toceranib phosphate, piroxicam, and propranolol. Although only a small, cautery‐affected biopsy sample could be obtained – preventing definitive histopathologic or immunohistochemical confirmation – the patient demonstrated marked tumour regression within 2 months and no radiographically detectable mass by 7 months. Treatment was well tolerated, even in the context of ACVIM Stage B2 myxomatous mitral valve disease.

The magnitude and durability of the response exceed typical outcomes reported for unresectable visceral HSA and raise the possibility of a beneficial interaction among tyrosine kinase inhibition, COX inhibition, and β‐adrenergic blockade. However, this interpretation remains speculative given the diagnostic constraints, lack of molecular characterisation, and reliance on radiography and ultrasonography – rather than advanced cross‐sectional imaging – for follow‐up.

Despite these limitations, the present findings offer preliminary support for considering combined toceranib, piroxicam, and propranolol as a potential therapeutic option in select cases of unresectable presumptive HSA. Prospective studies involving larger cohorts, standardised dosing regimens, and incorporation of molecular and imaging biomarkers will be essential to determine the reproducibility, safety, and broader clinical applicability of this multimodal approach.

## Author Contributions

Conceptualisation/methodology: W.D.P. Investigation/resources: W.D.P. Writing – original draft: W.D.P. Writing – review & editing: W.D.P. Visualisation: W.D.P. Supervision/project administration: W.D.P.

## Funding

The author has nothing to report.

## Ethics Statement

All procedures were performed as part of routine clinical diagnosis and treatment and followed institutional standards of care and the journal's animal ethics policy.

## Consent

Client informed consent was obtained for diagnostic imaging, surgery, off‐label medical therapy, and publication of this case report and accompanying images.

## Conflicts of Interest

The author declares no conflicts of interest.

## Data Availability

All data supporting the findings are included in the article and its figures. Additional de‐identified imaging files are available from the corresponding author upon reasonable request.
